# There’s Plenty of Room Right Here: Biological Systems as Evolved, Overloaded, Multi-Scale Machines

**DOI:** 10.3390/biomimetics8010110

**Published:** 2023-03-08

**Authors:** Joshua Bongard, Michael Levin

**Affiliations:** 1 Department of Computer Science, University of Vermont, Burlington, VT 05405, USA; 2 Allen Discovery Center at Tufts University, 200 Boston Ave., Suite 4600, Medford, MA 02155, USA

**Keywords:** biology, computer science, robot, artificial life, artificial intelligence, machine learning, evolution

## Abstract

The applicability of computational models to the biological world is an active topic of debate. We argue that a useful path forward results from abandoning hard boundaries between categories and adopting an observer-dependent, pragmatic view. Such a view dissolves the contingent dichotomies driven by human cognitive biases (e.g., a tendency to oversimplify) and prior technological limitations in favor of a more continuous view, necessitated by the study of evolution, developmental biology, and intelligent machines. Form and function are tightly entwined in nature, and in some cases, in robotics as well. Thus, efforts to re-shape living systems for biomedical or bioengineering purposes require prediction and control of their function at multiple scales. This is challenging for many reasons, one of which is that living systems perform multiple functions in the same place at the same time. We refer to this as “polycomputing”—the ability of the same substrate to simultaneously compute different things, and make those computational results available to different observers. This ability is an important way in which living things are a kind of computer, but not the familiar, linear, deterministic kind; rather, living things are computers in the broad sense of their computational materials, as reported in the rapidly growing physical computing literature. We argue that an observer-centered framework for the computations performed by evolved and designed systems will improve the understanding of mesoscale events, as it has already done at quantum and relativistic scales. To develop our understanding of how life performs polycomputing, and how it can be convinced to alter one or more of those functions, we can first create technologies that polycompute and learn how to alter their functions. Here, we review examples of biological and technological polycomputing, and develop the idea that the overloading of different functions on the same hardware is an important design principle that helps to understand and build both evolved and designed systems. Learning to hack existing polycomputing substrates, as well as to evolve and design new ones, will have massive impacts on regenerative medicine, robotics, and computer engineering.

## 1. Introduction

**Figure 1:**
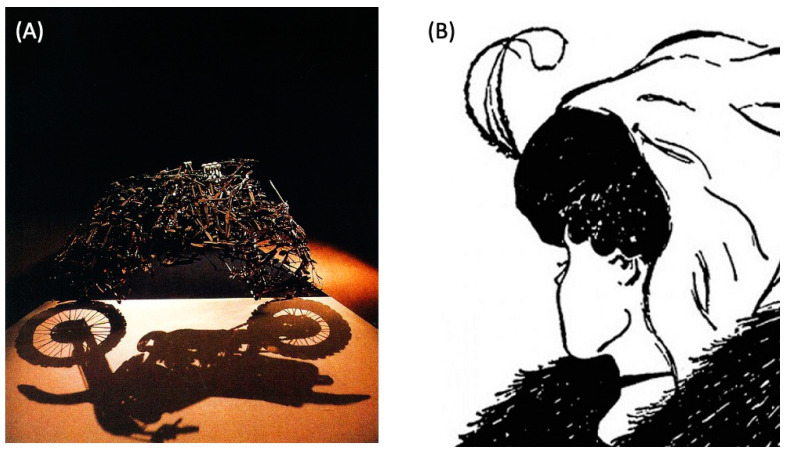
Polycomputing concepts in art. (**A**) Sculpture by Shigeo Fukuda, “Lunch with a helmet on”, 1987—appears as a random pile of knives and forks but when observed in just the right way, light moving through the sculpture reveals another pattern (a motorcycle) present at the same time in the same structure. (**B**) A well-known bistable (ambiguous) image, “My Wife and my Mother-in-Law” by British cartoonist William Ely Hill in 1915, reveals how our nervous system is not suited to taking in multiple meanings—it prefers to squash down to a single interpretation, even if it then has to vacillate back and forth.

In Feynman’s famous lecture titled “There’s Plenty of Room at the Bottom” [1], he argued that vast technological progress could be achieved by learning to manipulate matter and energy at ever-smaller scales. Such potential could presumably be exploited by natural selection as well. How does biology expand the adaptive function of an existing system? It cannot go down, since there is already something there, exhibiting functional competencies at every level [2]. Instead, it squeezes more action from each level by overloading mechanisms with multiple functions—which we term as polycomputing. We argue that the most effective lens for a wide range of natural and engineered systems must enable a multiple-observers view where the same set of events can be interpreted as different computations (Figure 1 illustrates how artists have recognized this feature). Indeed, depending on their definition of computation, some human observers may conclude that the observed system is not computing at all.

Herein, we review remarkable examples of biological polycomputing, such as spider webs that serve as auditory sensors and prey capture devices [3], and holographic memory storage in the brain [4,5]. We will also review emerging examples in computer and materials engineering [6]. We provisionally define polycomputing as the ability of a material to provide the results of more than one computation in the same place at the same time. To distinguish this from complex materials that necessarily produce complex results in the same place at the same time, such as the multiple peaks in the frequency spectrum of a vibrating material, polycomputing must be embodied in a material that has been evolved, or can be designed to produce particular results—such as the results of particular mathematical transformations like digital logic—and must be readable by other parts of the material or other devices. That is, the computation, to be considered a computation, must be useful to one or more observers (which, in biology, can exist on multiple scales, with multiple subsystems from the molecular to the whole organism, or swarm levels being able to reap the diverse evolutionary benefits of a single process if they interpret it as processing information that provides an adaptive advantage). These ideas, which describe new ways of understanding and exploiting polycomputing in biology, may suggest ways to improve synthetic polycomputing systems, which, in turn, will shed light on the nature of computation, evolution, and control. Biological systems that polycompute also contribute to an ongoing conceptual debate within interdisciplinary science—the applicability of computer frameworks and metaphors to living systems [7]—in three ways. First: if polycomputing changes our understanding of what computation is, that might change whether we consider a living system to be a computer (Section 1.1). Second: a living system (or inorganic material) may be considered to be polycomputing, depending on one’s point of view, suggesting that observer dependence is unavoidable when considering whether or what a living or engineered system computes (Section 1.2). Third: increasingly intricate admixtures of technological and biological components that compute are forcing a redefinition of life itself (Section 1.3).

### 1.1. What Constitutes a Computer?

The notion of a “computer” needs to be expanded: it no longer only refers to the sequential, deterministic, silicon-embodied, human-programmed, von Neumann/Turing architectures with which biologists are familiar. Those are indeed dissimilar to living systems. There is now a widening array of computational substrates and robots that are often massively parallel (such as GPUs and computational metamaterials [8]), stochastic (hard to predict) [9], able to exploit non-obvious (and potentially not-yet-understood) properties of the exotic substrates they are built from [10], emergent, produced by evolutionary techniques [11], and built by other machines [12] or programmed by other algorithms [13,14,15]. The benefit of considering biological systems as members of this broader class is that it avails powerful conceptual frameworks from computer science to be deployed in biology in a deep way, and therefore to understand life far beyond its current limited use in computational biology. Moreover, exploring this powerful invariant between natural and synthetic systems can enrich intervention techniques within biology and improve the capabilities of engineered devices, revealing gaps in our understanding and the capabilities of both computer science and biology. Polycomputing is a powerful but, as of yet, under-appreciated example of the many ways in which the wider class of computer devices can help to revolutionize the life sciences. In the same way that organic and inorganic materials acting as computers increasingly challenges the claim that living materials are not computers, we have argued elsewhere [16] that the widening array of materials that can now be viewed or engineered with as machines is corroding the classic claim that living systems are not machines, and forcing an improved definition of “machine” that escapes the narrow definitions of past decades, which are no longer appropriate [17,18,19].

### 1.2. Observer Dependency

In the statement “living things are (or are not) computers”, “are” implies the existence of an objective, privileged view of both computers and biology that allows an unambiguous, universal decision as to whether they are related. This binary view is untenable and gives rise to numerous pseudo-problems. We argue instead for an observer-dependent view, in which computational formalisms are just metaphors; of course, *all* scientific concepts are just metaphors, with varying degrees of utility (which is not binary). Once we come to grips with the fact that “all models are wrong but some are useful” [20], it is possible to adopt a pragmatic approach [21] in which anything is a computer in a given context, to the degree to which it enables an observer to predict and control that thing better than any competing metaphors allow us to do. In this view, whether something is computing is not a philosophical question, but one to be settled experimentally by specifying a computational framework and showing empirically what new levels of capability, experiments, and research are enabled by adopting that framework. The only thing left is to enable system subcomponents, not just human scientists, to act as observers [22,23,24,25]. From that perspective, the quality of a computational metaphor in science is evidenced by its degree of productivity in new experimental capabilities, while the quality of a computational stance adopted by a biological subsystem is cashed out by the adaptive advantage that is evinced by it. Of course, it is expected that future progress will uncover even better frameworks, so the answer is never final, but always provisional and relative to a specific perspective. This view is akin both to the intentional stance in the philosophy of the mind [26], and in the driving of the development of frameworks and tools from cognitive science that can be broadly deployed across biology and the biomedical sciences [2,27,28].

### 1.3. What Things Are Alive?

Finally, the question of what constitutes a “living thing” is itself undergoing a renaissance due to the new chimeric, synthetic, and bioengineering techniques being developed [29]. Active matter, synthetic biology, and biohybrids [30,31,32,33,34,35,36] are blurring the line between evolved and designed systems, and dissolving the distinctions between “life” and “machine” [16,18,37], which were easy to maintain when our capabilities did not permit the construction and analysis of the full option space of agents [38,39]. At this point, the life sciences have expanded well beyond the N = 1 example of phylogenetic history here on Earth, to a clear mandate to understand life as it can be via synthetic and exobiological explorations [40,41,42,43,44,45,46].

### 1.4. From a Philosophy to a Science of How Life (Poly)Computes

We propose that the way to side-step philosophical debates about whether biological systems “are” computers is to adopt an observer-centered, scale-free view of the computational formalisms in biology. Polycomputing is an ideal example of a linking concept that will enrich both fields, which enables a number of fascinating questions with many fundamental and practical implications to be asked. What are the key functional and control properties of polycomputing systems? How does evolution create systems where multiple functions reside in the same hardware, and what does this design principle mean for evolvability? How can we derive intervention policies that make rational changes in existing polycomputing systems, and what are the efficient paths to the design of novel polycomputing materials, control algorithms, and device architectures?

Regardless of whether or not a living system is distally observed, it still polycomputes, because life itself adopts the same operator-dependent approach. In other words, a biological mechanism polycomputes because its functionality and signaling are interpreted in different ways by other components of that same living system. Each level and component of a living system are simultaneously observers and hackers, interpreting and taking advantage of different aspects of the mechanisms in their microenvironments, in parallel. Life polycomputes because it is a set of overlapping, competing, cooperating nested dolls, each of which is doing the best it can to predict and exploit its microenvironment [47,48,49,50,51,52,53].

### 1.5. Why “Life as Computation” Matters

The transfer of knowledge between the disciplines of biology and computation forms a positive feedback loop for increasing the insight within both. Biological examples help to widen the range of implementations for computing devices and provide novel ideas for architectures [54,55,56,57,58]; unconventional computing platforms include fungal networks, ant colonies, and DNA. In complement, computer science and its idea of functionalist substrate independence (multiple realizability) helps biologists to focus on essential, rather than contingent, design principles, expanding biology beyond zoology and botany. This has been most prevalent in neuroscience [59,60,61], but more recently has been extended far beyond it, in recognition of the fact that neural dynamics are only an extension of far older biological problem solving architectures [28,62,63,64].

A key conceptual insight from computer science that informs biology concerns the nature of computation. For example, the field of physical reservoir computing [65], in which a neural network is trained to map the dynamics occurring within an inorganic, biological, or technological system (the “reservoir”) into an output desired by a human observer, helps us to see the observer-dependent aspect of biology. This offers ways to think about biology as nested societies of elements which are exploiting the information-processing capacities [66] of their living environment. Cells, parasites, conspecifics, commensal organisms, and evolution itself are all hackers in the sense of using their parts and neighbors as affordances in whatever way they can, rather than in some single, unique, privileged, and objective way that reflects “true” functionality.

The concepts of superposition in quantum mechanics and the primacy of observer frames in relativity have transformed the understanding of this phenomena on very small and very large scales, respectively. Polycomputing challenges us to apply the same concepts to computation and life at mesoscales. Here, we overview the concepts of superposition and observer frames as they are applied to mesoscales and argue that the polycomputing lens, like the agential matter lens [67,68], helps us to understand, predict, and control new classes of evolved and designed materials, with numerous applications ranging from regenerative medicine to engineering.

## 2. Current Debates: Dissolving Dichotomous Thinking

Whenever technological progress within a particular domain begins to slow, researchers often look to nature for fresh inspiration. Examples of this include the use of photosynthesis for new energy capture devices [69] and flapping wings for new drone designs [70]. Following this tradition, the increasing difficulty of packing more computing ability into microchips [71] challenges us to seek new paths forward by considering how computation is embedded within living systems. Comparing how organisms and machines compute requires one to view an organism as a kind of machine; otherwise, no comparison is possible. The debate about how or whether organisms are machines has a long history, and has become more intense in recent years [16,17,18,37,62,72,73], as various disciplines not only compare life to machines, but attempt to merge the two (reviewed in [38]).

Our usage of the term “machine” in what follows will denote a subset of machines that are capable of computation. Such machines include robots and physical computers but exclude simple mechanical devices such as combustion engines and flywheels, for which no way to stimulate or exploit them to produce computation has yet been invented (if such interventions are discovered, these technologies can then be considered as belonging more to the class of computational machines). In the spirit of our thesis, we acknowledge that there is no clear dividing line between these two “types” of machines, as circuitry-free machines such as mechanical computers, physical reservoir computers, [65] and computational metamaterials [8] can still compute. As always, there is a continuum: in this case, it is across machines capable of more or less computation. A possible exception may exist for machines that compute by exploiting quantum effects, although even there the notion of an observer plays a key role in deriving binary outcomes from a fundamentally indeterminate reality. The usage of the term “machine” rather than “computer” in what follows is meant to remind the reader that we are considering organisms vis-a-vis human-made things that compute, rather than just comparing them to traditional computers.

### 2.1. Structure Function Mapping and Polycomputing

An obvious starting point for the comparison between organisms and computers, or organisms and machines, is to assume a 1-to-1 mapping between the structure and function. A comparison can then be attempted between the organism’s and machine’s structures, and then between their functions. Finally, one can compare the structure-to-function mappings of the organisms and machines. However, teasing apart the structure and function for such comparisons is difficult. Genetics [74] and neuroscience [75] can both provide historical examples of how 1-to-1 structure/function mappings were rapidly replaced by models with increasingly dense and non-intuitive interactions between their structural and functional units. Even harder than making predictions based on this nontrivial structure-to-function mapping is inferring which interventions to make for rational changes at the system level, as is needed in the example of regenerative medicine—replacing complex organs such as hands and eyes [27,28]. Advances in other areas where biology and computer science meet are similarly demolishing these long held dichotomies (Table 1).

Indeed, an understanding of this wide range of implementations (materials, including organic components) and origin stories (e.g., evolutionary design techniques [76]) for machines makes it clear that, in many cases, a modern machine lens for life facilitates progress. The machine metaphor is a functional approach that seeks to develop possible efficient ways to predict, control, communicate with, and relate to a system and its reliable behavior modes. However, one aspect has lagged in both engineering and biology. It is relatively easy to see that technological or living components can support different functions at the same time but at different spatial scales: myosin, for example, supports muscle fiber contraction and legged locomotion simultaneously. It is also easy to see how components can support different functions on the same spatial scale but at different times: myosin can support legged locomotion and then tree climbing. However, it can be difficult to see how a component can provide multiple uses for multiple beneficiaries (or compute different functions from the vantage point of different observers) on the same spatial scale and at the same time. Investigating this last phenomenon—polycomputing—enables not only a new set of questions for biology, but also a quest for engineers to understand how to pack more functionality into the same machine.

**Table 1:** Some common assumed distinctions in biology and technology, and recent advances that serve as counterexamples, suggesting a spectrum of complementarity.

Assumed Distinction	Counterexamples
Software/Hardware	Physical materials that compute [65] and learn [77].
Tape/Machine	Tape-less von Neumann self replicators [78] (assuming a Turing machine architecture)
Digital/Analog	Evolved digital circuits can exploit electromagnetic properties of the circuit’s substrate [11].
Machine/Life form	AI-designed organisms [78,79].
Automaton/Free agent	The intentional stance [26].
Brain/Body	Computational metamaterials [8].
Body/Environment	Other cells are the environment for a cell in a multicellular body.
Intelligent/Faking it	AI technologies that seem to pass verbal [80], visual [81], or physical [82] Turing tests.
Made/Evolved	Artefacts designed by human-created evolutionary algorithms.

### 2.2. Dichotomous Thinking in the Life Sciences

Biology does not really support dichotomous categories. While it is sometimes convenient for biologists to adopt discrete criteria for specific characters, evolution and developmental biology both exhibit remarkable examples of scale invariance and gradual change. Neither process supports any kind of clean bright line that separates the cognitive human being from the “just physics” of a quiescent oocyte or the “true grounded knowledge” from the statistically driven speech behavior of babies and some AIs, etc. (Table 1). All of these, like the process of slowly changing a being from a caterpillar to a butterfly [47], show that familiar categories in fact represent the poles of a spectrum of highly diverse mixed properties. The interoperability of life [47,83,84,85] enables chimeras at all levels of an organization, which provide a continuum of every possible combination of features from supposedly distinct categories (Table 1), making it impossible to objectively classify either natural or artificial chimeras [29,38]. It is becoming increasingly apparent that the departmental, funding, and publication distinctions between disciplines (e.g., neuroscience and cell biology, are much more of a practical consequence of our cognitive and logistical limitations than the reflection of a deep underlying distinction. In fact, these divisions obscure important invariants: the symmetries across categories that enable unifications, such as the use of cognitive neuroscience techniques to understand the collective intelligence of cells during morphogenesis [27,28,86,87], or indeed of physics itself [24,88].

### 2.3. Dichotomous Thinking in Computer Science

Advances in the computational sciences also increasingly disrespect human-devised categorical boundaries. One such boundary under attack is that between the body and brain. One set of technologies that is eating away at this distinction is physical computing; a conceptual advance doing similarly caustic work is that of morphological computation. In mechanical computing, computation is performed without recourse to electronics and instead relies on optical [89], mechanical [90], or quantum [91] phenomena. Recent advances in mechanical computing show how inert bulk materials can be induced to perform non-trivial calculations, including error backpropagation, the algorithmic cornerstone of modern AI [77]. A recent demonstration by one of the authors (Bongard), showing that propagation of acoustic waves through granular metamaterials can be exploited to perform multiple Boolean operations in the same place at the same time [8], can be considered to be the first example of mechanical polycomputing. Mechanical computing, and now, mechanical polycomputing, challenge the assumption that, in organisms, there needs to be one subsystem that computes and controls (such as the nervous system) and another that accepts that control (the body).

Morphological computation, a concept originating in the robotics literature, upholds that the body of an animal or robot can indeed compute, and, moreover, it can “take over” some of the computation performed by a nervous system or robot control policy [92,93]. Although mechanical computing and morphological computing are similar in spirit, in mechanical computing, the bulk material passively accepts whatever computation is forced upon it. In contrast, in morphological computation, the animal or robot may adaptively perform computation either neurally or mechanically, depending on the current demands of its environment. This flow of computation back and forth between the body and brain (or between the digital circuitry and bulk materials) suggests that the two human-devised categories of the “body” and the “brain” should not be as distinct as once thought.

### 2.4. Polycomputing in Bodies and Brains

If polycomputing is to be considered a kind of computation, one can then ask whether polycomputation can be shuttled back and forth between biological bodies and brains, or if it can be made to do so between machine bodies and brains. For this to work, polycomputation must be implementable in different kinds of substrates. Traditional computation is assumed to be substrate agnostic: if it is configured appropriately, any physical material can compute. In contrast, only vibrational materials have been shown capable of polycomputing to date, as polycomputation requires the storage of the results of multiple computations at the same place and at the same time, but at different peaks in the frequency spectrum (non-vibrational materials may also be capable of polycomputing: materials with vectorial modes could store multiple results in different vector elements, or multimodal systems could store results in different modes). This focus on vibration would seem to preclude some materials, such as digital circuitry and biological nervous systems, from performing polycomputation, since digital circuitry traffics in electrons, and nervous systems traffic in chemicals and ions, while neither seem to traffic in vibration. At first glance, this seems poised to rescue the brain/body distinction via the surprising route of suggesting that bodies and brains are different things, because bodies polycompute, but brains do not.

However, this odd-seeming distinction may be short-lived. It has been shown that neurons may communicate mechanically [94] in addition to electrically and chemically. If so, such mechanical neural communication may contain vibrational components, suggesting that nervous systems may be polycomputing as well. If this turns out to be the case, it, in turn, opens up the possibility that nervous tissues may have evolved incremental enrichments of the non-neural cells’ already-proven ability to polycompute. This would once again frustrate our attempts to cleave the body from the brain, in this case by the claim that one polycomputes, while the other does not.

Mechanical computing and morphological computation are closely related to another way in which computer science provides useful viewpoints for biology. In computer science, the view that an algorithm *drives* (functionally determines) outcomes, even though it is implemented by the microphysics of the electron flows through a CPU, is accepted and indeed essential to performing the useful and powerful activity of programming. This is in stark contrast to the debates within biology and neuroscience about whether higher levels of description are merely epiphenomenal [95,96,97,98,99], supervening on biochemical microstates (reductionism). Computer science clearly shows how taking a causal stance at higher levels enables progress. Indeed, the recent advances in information theories around quantifying causal emergence [99,100] show how the same Boolean network can compute different functions simultaneously, depending on the level of analysis chosen by an observer [99]. This has interesting biological implications, since such networks are a popular model for understanding the functional relationships between genes [101,102,103].

Biological nervous systems—the human brain in particular—have attracted increasingly computational metaphors throughout the industrial revolution and information age. The application of computational concepts to brains has had unintended consequences, most of all being the implicit assumption that tissues, cells, and other biological systems that are not brains do not compute. However, the brain–body dichotomy is being increasingly dismantled by studies of basal cognition (i.e., intelligence in unfamiliar embodiments) in plants [104,105], single somatic cells [106,107,108], microbes [109,110,111,112], and at the tissue level in organisms [113,114,115,116]. Indeed, the bioelectric and neurotransmitter dynamics that implement predictive processing and other computations in brains are speed-optimized versions of the extremely ancient bioelectrical computations that navigated spaces (such as anatomical morphospace and physiological space, etc.) long before brains and muscles appeared [64,117,118]. Indeed, the tools of neuroscience—from conceptual approaches such as active inference [28,87] to molecular tools such as optogenetics [119,120,121,122]—do not distinguish between neurons and non-neural contexts, being broadly applicable across biology.

The benefit of dissolving these arbitrary distinctions is that commonalities and fundamental design principles across substrates are beginning to emerge across evolved and designed constructs on all scales [38,107,123]. Frameworks that are to survive the next decades, in which technological advancement will further enmesh biology and technology, must facilitate experimental progress at the expense of philosophical preconceptions. More than that, they must provide a unifying insight by identifying the symmetry and deep order across the fields to combat the ever-growing problems of big data and the interpretability crisis [124,125]. Here, we delve into one emerging principle: polycomputing, which places front and center the fascinating issues of form, function, control, interpretation, and the role of the observer.

## 3. Learning from Superposed Systems in Engineering

The ability to compute and store multiple results in the same locality at the same time is of obvious engineering interest, as it could greatly increase computational density. Various technologies are now being built that roll back the assumption that such superposition is impossible. These technologies, reviewed below, suggest ways of looking for similar phenomena in natural systems.

Quantum computing has made it clear that multiple computations can be performed simultaneously. However, practical and general-purpose quantum computing remain a distant prospect. Recently, one of the authors (Bongard) showed that quantum effects are not necessary for polycomputing [8]: even relatively simple materials that are composed of only 30 parts are capable of computing two Boolean logical functions (AND and XOR) in the same place at the same time. This non-quantum form of computational superposition suggests not only that more computation may be packed into smaller spaces, but also that the fundamental way in which computation arises in technological and biological materials may need to be rethought.

Holographic data storage (HDS; [126]) is another set of related technologies that do not assume that only one datum or computational result is stored locally. HDS stores and reads data that have been dispersed across the storage medium. It does so by etching a distributed representation of a datum across the storage medium, for example with laser light, from a particular direction. That datum can then be retrieved by capturing the reflection of light cast from the same direction. By storing data in this way, from multiple directions, parts of multiple pieces of data are stored in the same place, but accessed at different times. Exactly how this can be achieved in hardware, such that it affords appreciable increases in the storage density more than current traditional approaches, has yet to be resolved.

A third technology relaxing the assumption of the data/compute locality is physical reservoir computing (PRC). PRC, inspired by HDS, attempts to retrieve the results of the desired computations by exciting inert bulk materials, such as metal plates or photonic crystals, and capturing the resulting mechanical vibrations or refracted light, respectively. Different computations can be extracted from the same material by exciting it in different ways. An attempt to “program” PRCs, thus easing the ability to extract the desired computation from them, has also been reported [127]. Notably, this method has been used to create “deep physical neural networks” [77]: the input, and the parameters describing an artificial neural network, are combined into forces that are supplied to the material. The forces captured back from the material are interpreted as if the input had been passed through a neural network with those parameters. The errors in the output can then be used to modulate the input, and the process repeats until a set of input forces has been found that produces the desired output. Importantly, the internal structure of the bulk material is not changed during this training process. This means that the same material can embody different computations. Just how distributed or localized these computations are within these materials remains to be seen.

Other materials are not only changed by the forces acting on them, but retain an imprint of those forces even after they cease: they are capable of memory. Efforts are also underway to design such materials to maximize the number of overlapping memories that they can store [128,129]. The ability of the designed materials to absorb the forces, compute with them, and output the transformed forces that encode the results of those computations, holds great promise for robotics. If future robots can be built from such materials, the force propagation within them could simultaneously produce external behavior and internal cogitation, without requiring distinct behavior-generating components (the body) and computation-generating components (the brain). Indeed, soft robots are already demonstrating how exotic materials enable the traditionally distinct functions of sensation, actuation, computation, power storage, and power generation to be performed simultaneously by the same parts of the robot’s body [130].

## 4. Biology Is Massively Overloaded: Polycomputing

**Figure 2:**
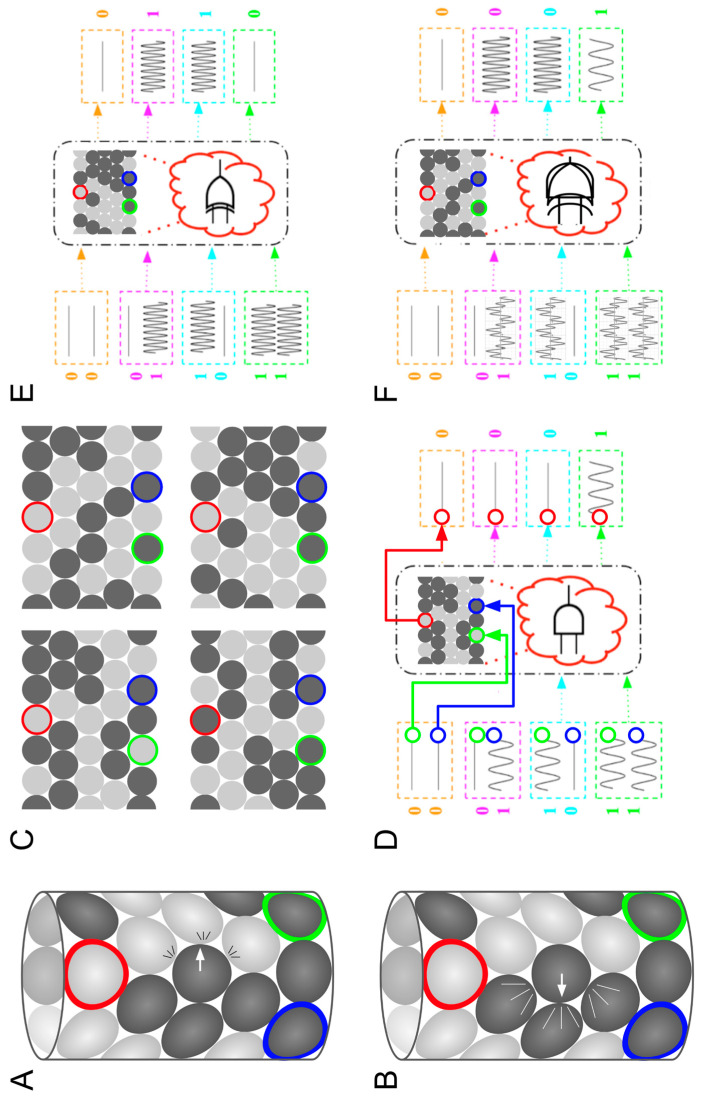
Engineering polycomputing materials. (**A**) A granular metamaterial can be assembled by embedding stiff (dark gray) and soft (light gray) particles into a sheet and wrapping it into a tube. If a particle collides with soft particles, it only slightly affects their motion. (**B**) If it hits rigid particles, their motion is affected more. (**C**) An evolutionary algorithm (EA) can be created that evolves populations of metamaterials, where each one has a unique combination of stiff and soft particles. The EA can then delete those metamaterials that perform poorly at some desired task, such as performing a computation, and make randomly modified copies of those that do a better job. (**D**) This can result in the evolution of a material that acts as an AND gate, a building block of computers: some designated ‘output’ particle (red) should only vibrate if two other ‘input’ particles are vibrated from outside the system (green and blue). (**E**) An evolutionary algorithm can be asked to evolve a metamaterial that acts as an AND at one frequency, but also to act as another computational building block, an XOR gate, at a higher frequency: the output particle should only vibrate if one of the input particles is vibrated. (**F**) This process results in the evolution of a polycomputing material: if inputs are supplied at two different frequencies, the evolved material acts as an AND and XOR gate simultaneously: it provides the results of these two computations at the same place at the same time (the output particle), but at different frequencies. Details can be found in [8].; panels (**D**–**F**) used with permission from ACM.

Analyzing natural systems to determine whether or how they perform polycomputation is particularly challenging, as most analytic approaches are reductionist: they “reduce” to characterizing one phenomenon that arises in one place, at one time, under one set of circumstances. Synthesis is also difficult: polycomputable technologies seem, to date, resistant to the traditional engineering design principles such as hierarchy and modularity. A fundamental problem is that typical human designs are highly constrained, such that any changes made to optimize one function often interfere with another. Although humans struggle to manually design polycomputing technologies, it turns out that AI methods can do so, at least in one domain. We have recently applied an evolutionary algorithm—a type of AI search method—to automatically design a granular metamaterial that polycomputes. It does so by combining vibrations at different frequencies at its inputs, and providing different computations in the same place, at the same time, at different frequencies. Figure 2 illustrates this process.

Many biological functions have been usefully analyzed as computations [62] (Table 2). These include molecular pathways [128,131], individual protein molecules [63], cytoskeletal elements [129,132,133], calcium signaling [134], and many others. Although differing from the most familiar, traditional algorithms, the massively parallel, stochastic (indeterministic), evolutionarily shaped information processing of life is well within the broad umbrella of the computations familiar to workers within the information sciences. Indeed, information science tools have been used to understand cell- and tissue-level decision making, including estimations of uncertainty [108,135,136,137,138,139,140,141,142,143,144,145], analog/digital dynamics [146], and distributed computations [104]. Bioelectric networks within non-neural tissues, just like their neural counterparts, have shown properties that are very amenable to polycomputation, including the ability to store diverse pattern memories that help to execute morphogenesis on multiple scales simultaneously [28,30,116,147,148,149,150,151,152,153], and enable the same genome to produce multiple diverse outcomes [154].

A key aspect to recognizing unconventional computing in biology is that the notion of “what is this system *really* computing” has to be dropped (because of the multiple observers issue described above; see also [155] for a discussion of the role of the observer in unconventional computing). Once we do this, biology is rife with polycomputing on all scales. An example of this includes the storage of a (very large) number of memories in the same neuronal real-estate of the brain [156,157], and many others are summarized in Table 2. We do not yet know whether the prevalence of polycomputing is because of its efficiency, robustness, or other gains that override the evolutionary difficulty of finding such solutions. Or, perhaps we overestimate this difficulty, and evolution has no problem in identifying such solutions—they may indeed be the default. If so, it may be because of the generative, problem solving nature of developmental physiology that is the layer between the genotype and the phenotype [38,158]. For these reasons, polycomputing may join degeneracy and redundancy [159], as well as the minimization of stress and frustration [2,160,161,162,163], as one of the organizing principles that underlies the open-ended, robust nature of living systems.

**Table 2:** Examples of biological polycomputing at diverse scales.

Multiple Computations in the Same Biological Hardware	Reference
Mitochondria also act as micro-lenses in photoreceptors	[164]
Proteins acting in multiple (fluctuating) conformations	[165]
Pathways and transcriptional networks regulating real-time physiology and performing learning at the same time	[161,166,167,168,169,170,171,172]
Gene regulatory networks with multiple memories/behaviors	[170,172,173,174]
Chemical networks performing neural network tasks	[171,175]
RNA encoding enzyme and protein functions	[176,177,178,179]
ATP as an energy source and neurotransmitter	[180]
DNA with more than one active reading frame (overlapping/dual-coding genes)	[181,182]
Ion channels that are also transcription factors	[183]
DNA transcription factors working in DNA replication machinery	[184]
Polysemanticity and superposition of meaning in neural networks and language understanding	[185,186,187]
Cytoskeleton performing computations via simultaneous biomechanical, bioelectrical, and quantum-mechanical dynamics	[188,189,190,191,192,193,194,195,196,197]
Electrophysiological networks performing memory functions while regulating heartbeat	[198,199,200]
Bioelectric networks performing physiological functions while also regulating morphogenesis	[117]
Spider webs as auditory sensors and structural elements	[3]
Pleiotropy: most genes have multiple functions	[74]
Holographic memory in the brain	[201]
Multiple behaviors in the same neuronal circuit	[202]
Multiple personalities in the same brain (dissociative identity disorder and split brain studies)	[203,204]
Calcium dynamics performing as a hub in a huge bowtie network of diverse simultaneous processes	[205,206]

### 4.1. Evolutionary Pivots: Origins of Polycomputing?

**Figure 3:**
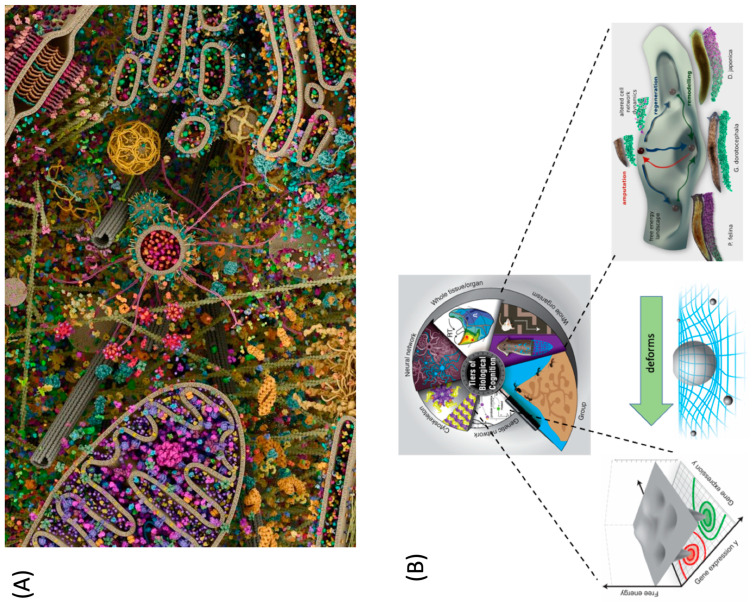
Polycomputing architectures in biology. (**A**) 3D computer rendering of a eukaryotic cell modeled using X-ray, nuclear magnetic resonance (NMR), and cryo-electron microscopy datasets for all its molecular actors. The image was created by Evan Ingersoll and Gaël McGill (Digizyme Inc.) and illustrates the pressure on biology to have each component perform multiple duties (there is not much room to add additional components); this image shows a dilute cytoplasm relative to a real cell). Used with permission. (**B**) Multi-scale competency architecture of life consists of molecular networks which make up cells, which make up tissues, which make up organs, which make up organisms within swarms. Each layer is performing specific functions simultaneously; for example, the tissue layer is attempting to compute the correct attractor for the collective morphogenetic behavior of planarian fragment cells, which can build one of several head shapes). Each layer deforms the action landscape for the layer below it, providing incentives and shaping geodesics that force the lower-level components to use their behaviors in service of the higher level’s goals. Taken with permission from [22]. Images in panel B by Jeremy Guay of Peregrine Creative Inc. and Alexis Pietak.

Evolution is remarkably good at finding new uses for existing hardware due to its fundamental ability to generate novelty, in order to exploit new niches while being conservative in terms of building upon what already exists. This ability to simultaneously innovate and conserve plays out across structural, regulatory, and computational domains. Moreover, the re-use of the same conserved mechanisms in situ has enabled evolution to pivot successful algorithms (policies) from solving problems in metabolic spaces to solving them in physiological, transcriptional, and anatomical (morphospace) spaces, and finally, once muscles and nerves arrived on the scene, to 3D behavioral spaces [22,28,64,207]. For example, [64], the same ion channels that are used for the physiological control of cell homeostasis and metabolism are used *simultaneously* in large-scale bioelectric circuits that compute the direction of the adaptive changes in growth, and form in embryogenesis, metamorphosis, regeneration, and cancer suppression [64,112,117,208,209,210,211,212]. Indeed, in some animals such as planaria and axolotl, this all happens at the same time, as these exact same mechanisms in neural cells are guiding behavior [47]. Biology extensively uses polycomputing because it uses a multi-scale competency architecture, where every level of the organization is competent in solving certain problems within its own space. It is doing so at the same time via the same physical medium that is interpreted by observers on different scales, which exploits the results of those computations [2,22] (Figure 3).

Polycomputing is seen even at the lowest scale of molecular biological information. It has long been known that genomes are massively overloaded, providing polycomputing not only because of the multiple reading frames (overlapping genes) for some loci [181,182], but also because the question of “what is this gene for?” may have a clear answer at the molecular scale of a protein, but often has no unique answer at the phenotypic scale, because complex traits are implemented by many genes, and many (or most [74]) genes contribute to multiple mesoscale capabilities. Moreover, epigenetics enables the same genomic information to facilitate the embodied computation that results in multiple different anatomical, physiological, and behavioral forms [213,214].

### 4.2. Polycomputing and the Range of Phenotypic Possibility

**Figure 4:**
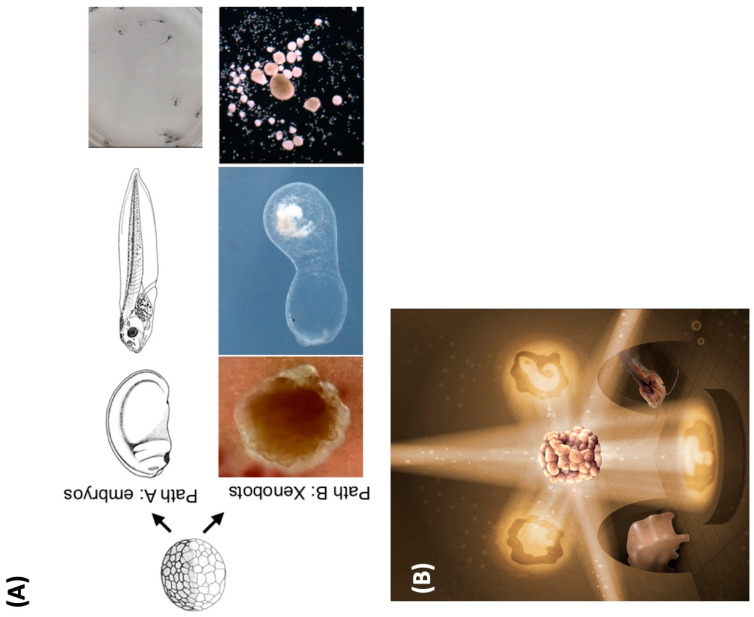
What does the Xenopus laevis genome specify? (**A**) The standard Xenopus laevis genome (in a frog egg) typically causes the construction of a set of standard embryonic stages (Path A) which results in tadpoles with specific behaviors. However, in a different context, the skin cells can autonomously create a Xenobot (Path B)—a spherical construct with autonomous motion, a different morphogenetic sequence, and behaviors such as kinematic self-replication. The same genomic information contains simultaneously seeds of emergent tadpoles or Xenobots. (**B**) Similar to the iconic cover image of the classic book Godel, Escher Bach [229] this image illustrates how the same hardware (the standard frog egg in the middle) can be used to generate diverse forms of living constructs. Different environments, external signals, and physiological events can coax diverse morphogenetic outcomes out of a constant biological information string (DNA). Images in (**A**) courtesy of Xenbase and Douglas Blackiston, Levin lab. Image in (**B**) by Jeremy Guay of Peregrine Creative.

How much actionable information on how to assemble an adaptive, behaving organism can be packed into the same genomic and physiological information medium? A recent example of massive phenotypic plasticity are Xenobots—proto-organisms that result from a rebooting of multicellularity with frog skin cells [79,215]. The Xenobots self-assemble as spheroids that are self-motile, exhibiting a range of autonomous behaviors, including highly novel ones such as kinematic self-replication: the ability, of which von Neumann famously dreamed, to assemble copies of themselves from material found in their environment [78]. In the case of Xenobots, this material is the dissociated cells that are introduced into their surroundings. A key point is that Xenobots are not genetically modified, and their novel functionality is implemented by perfectly standard frog cells. So, what did evolution learn [216,217,218,219,220] in crafting the *Xenopus laevis* genome and the frog eggs’ cytoplasmic complements? It was not just how to make a frog, it was how to make a system in which cells allow themselves to be coerced (by other cells) into a boring, two-dimensional life on the animal’s outer skin surface, or, when on their own, to assemble into a basal three-dimensional creature that autonomously explores its environment and has many other capabilities (Figure 4).

This capacity to do things that were not specifically selected for [221] and do not exist elsewhere in their (or others’) phylogenetic history reveals that evolution can not only create seeds for cellular machines that do multiple things, but for ones that can do novel things. This is because genomic information is overloaded by physiological interpretation machinery (internal observer modules): the exact same DNA sequence can be used by cells to build a tadpole or a Xenobot (and the same planarian genome can build the heads of several different species [222,223]). Thus, evolution teaches us about powerful polycomputing strategies because it does not make solutions for specific problems—it creates generic problem solving machines, in which the competition and cooperation of overlapping, nested computational agents at all levels exploit the ability of existing hardware to carry out numerous functions simultaneously. This is closely tied to recent advances at the interface of the fields of developmental biology and primitive cognition, with the generation of models in which larger-scale Selves (in psychological and anatomical spaces, etc.) arise as composite systems made of smaller Selves, all of which are pursuing diverse agendas [47,48,224].

**Figure 5:**
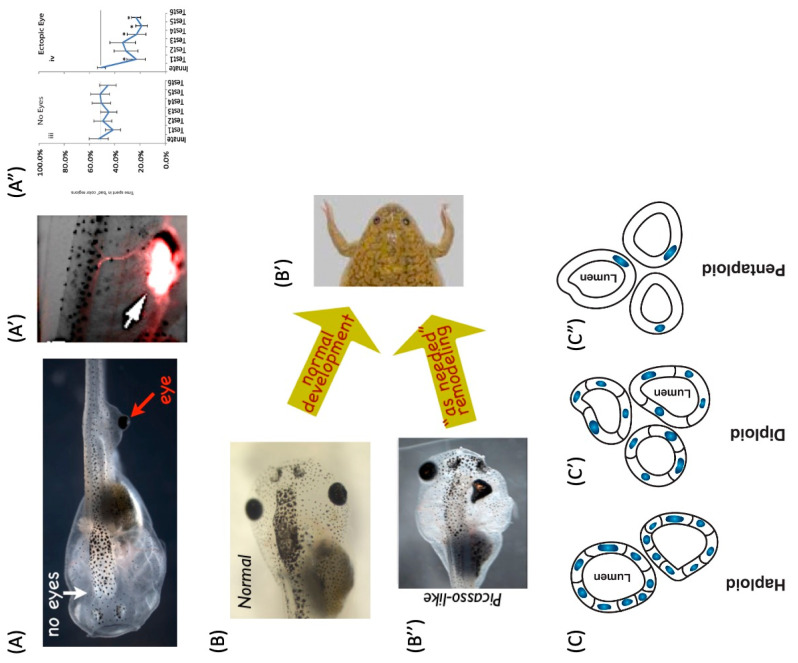
Morphogenesis plays the hand it is dealt. The essence of biological morphogenesis is that it does not assume much about current circumstances and attempts to create a viable organism with whatever is at hand [159]. Thus, frog embryos in which eye primordia cells are moved from the head to the tail still make good eyes (**A**), try to connect to the spinal cord (**A’**, red stain), and enable the organism to exhibit behavioral vision (**A”**), despite a completely novel visual system–brain architecture which had no evolutionary prep time to get used to the new arrangement—nothing needed to be changed (at the DNA level) to make this new configuration workable. Similarly, tadpoles (**B**) which must rearrange their face to turn into a frog (**B’**) can still do so even if everything is artificially placed in a scrambled configuration (**B”**), because each organ is able to move as needed to get its job done (reach a specific region of morphospace). Finally, the cross-level nature of this overloading of basic mechanisms is seen in newt kidney tubules schematized here in cross-section. While they normally consist of 8–10 cells that communicate to make a tubule (**C**), the cells can be made experimentally very large—in that case, fewer cells will work together to make the same size tubule (**C’**). In the case of enormous cells, a completely different mechanism (cytoskeletal bending) will be used by a single cell to create a lumen (**C”**)—showing how the same machine (genome and cell) can enable high-level anatomical goals to trigger diverse low-level molecular mechanisms, as needed for a global goal (such as reaching a specific region of anatomical morphospace). Panels (**A**,**A’**,**A”**,**B**) courtesy of Douglas Blackiston, used with permission after [230]. Panel (**B’**) courtesy of Erin Switzer, (**B”**) taken with permission from [231]. Panels (**C**,**C’**,**C”**) by Jeremy Guay of Peregrine Creative. * *p* < 0.05 (**A”**).

As surprising as these examples are, we should have already seen this coming—biology has to work like that, and it could not work otherwise. First, the “sim-to-real gap” (the difference an agent experiences when it is trained in virtual environments, built as a robot, and deployed into a real environment [225,226]) is as real for biology as it is for robotics: prior evolutionary experience in a past environment is not a reliable guide to the novel challenges that each generation experiences in new environments. Thus, evolution does not overtrain on prior examples, but generalizes, producing substrates that can compute different functions for different needs (Figure 5). Second, the evolutionary process is not working with a blank slate—the canvas of embryogenesis is made up of cells which used to be independent organisms. Evolution exploits the competencies of cells in numerous problem spaces as a toolkit of affordances to be exploited. Development is akin to behavior-shaping, where evolution finds signals that cells can send to other cells to push them into specific actions. This is a strong start for polycomputing as a design principle—working with an agential material [67] requires strategies that do not establish a single, privileged, new way of doing things, but instead drive adaptive outcomes by incentivizing subsystems to manage and exploit the many things that the material is already doing. This perspective is congruent with a “process metaphysics” [227,228]. Third, evolution simply would not work well with an architecture that did not support polycomputing, because each new evolutionary experiment would wreck the prior gains, even if it itself was an advance. Developing a new perspective on a set of events which provides a useful computational function enables subsystems to derive an adaptive advantage without having to change the events in question (thus not risking breaking something that other subsystems depend on). For example, we have shown that useful computational functions, such as associative memory, can be derived from a gene-regulatory network without changing the structure of the network (and thus without any possibility of adversely affecting any dependents), simply by a mechanism that interprets its outcomes in a particular way (by mapping specific nodes to the functional elements in an associative conditioning paradigm) [169,171]. This is readily evolved, and provides a way for evolution to squeeze additional benefits from the existing components without needing to change them in any way—all the work is done on the observer’s end, who also reaps the benefits without any negative consequences for the other internal observers (subsystems).

### 4.3. Evolving Polycomputing

The rate of evolution would be much slower without this multi-scale competency architecture—the ability of the parts to get their job accomplished even if circumstances change [159] In one remarkable example, the tadpole eye, placed in the wrong position on the head, or even on the tail [230], still provides vision, because the eye primordia cells can make an eye in aberrant locations, move it if possible [232], and if not, connect it to the spinal cord (rather than directly to the brain), providing visual signals that way [233,234]. This competency of the substrate in regulative development and remodeling [22,38] can neutralize the lethal side effects of many mutations, enabling the exploration of other possibly beneficial effects. For example, consider a mutation that causes the displacement of the mouth and also another effect, *E*, elsewhere in the body. The potential benefits of *E* might never be explored in a monocomputational developmental architecture, because the mouth defect would prevent the animal from eating and drive the fitness to 0. The exploration of the effect of *E* would have to wait for another mutation to appear that produces the same effect without untoward side effects elsewhere—a very long wait, and often altogether impossible. In contrast, in a polycomputing architecture, structures solve morphological and physiological problems simultaneously: the mouth will move to the right location on its own [232], in parallel to all of the other developmental events, enabling evolution to explore the consequences of *E*. Thus, the overloaded competencies of the cells and tissues allow for evolution to simultaneously explore the other effects of those mutations on a phenotype (of which pleiotropy is one example).

In this case, these competencies create the “hidden layer” of developmental physiology that sits between genomic inputs and phenotypic outputs, and provides a problem solving capacity: getting an adaptive task completed, despite changes in the microenvironment or in their own parts [2,22]. This occurs simultaneously at all scales of the organization (Figure 3), and thus, each level computes specific functions not only in its own problem space, but also participates in the higher level’s space (as a component) and has an influence that deforms the action space of its lower levels’ components [22]. By using behavior-shaping competent subunits as agential materials [67], evolution produces modules that make use of each other’s outputs in parallel, virtually guaranteeing that the same processes are exploited as different “functions” by the other components of the cell, the body, and the swarm.

The evolutionary pressure to make existing materials perform multiple duties is immense. However, much remains to be learned about how such pressure brings about polycomputing, and how some materials can be overloaded with new functions without negatively impacting the existing ones.

In parallel to such biological investigations, within the computer science domain, much work remains to be done to devise the optimization pressures that create polycomputing substrates, and then create new programming strategies that are suitable for polycomputing. For example, no programming language has yet been devised that truly takes advantage of the polycomputational metamaterials described above. Despite our ignorance about how evolutionary or optimization pressures can create polycomputational systems, what is clear is that evolution would not work at all if living things were not machines—predictable, tractable systems. The key aspects of machines are that they harness the laws of physics and computation, etc., in a reliable, rational manner to produce specific, useful outcomes. The evolutionary process exploits the fact that life is a machine by making changes to the material, the control algorithm, and indirectly, to the environment, in a way that gives rise to predictable, adaptive outcomes. Cells could not influence each other during development to reliably achieve the needed target morphologies if they could not be efficiently controlled. Life shows us the true power of the “machine”: a powerful, multi-scale polycomputing architecture, in which machines control and comprise other machines, all working at the same time in the same locality, but in different modalities and virtual problem spaces, producing massive amounts of plasticity, robustness, and novelty.

### 4.4. A New Approach to Identifying and Harnessing Computational Capabilities In Vivo and In Silico

One way to exploit this property is to use protocols that examine a particular mechanism for the novel things it can do, and for the best way to induce it to execute some of its capabilities. At the molecular level, an example is gene regulatory networks (GRNs), a formalism whereby a set of genes up- and down-regulate each other’s functions [235,236]. While GRNs and protein pathways are normally studied for ways to explain a particular aspect of biology (e.g., neural crest tissue formation or axial patterning in development [237,238]), we asked whether existing neural network models could have novel computational functions, specifically learning functions. Our algorithm took biological GRN models and, for each one, examined each possible choice of the triplets of nodes as the candidates for conditioned and unconditioned stimuli and response, as per Pavlovian classical associative learning [239]. We found numerous examples of learning capacity in biological networks and many fewer in control random networks, suggesting that evolution is enriching for this property [170]. Most strikingly, the same networks offered multiple different types of memory and computations, depending on which of the network’s nodes the observer took as their control knobs and salient readout in the training paradigm. This approach is an example of searching not for ways to rewire the causal architecture of the system for a desired function, but searching instead for a functional perspective from which an *unmodified* system already embodies novel functions.

This illustrates an important principle of biological polycomputing: evolution can prepare a computational affordance (the GRN) with multiple interfaces (different gene targets) through which engineers, neighboring cells, or parasites can manipulate the system to benefit from its computational capabilities. We suggest that this kind of approach may be an important way of understanding biological evolution: as a search for ways in which the body’s components can adaptively exploit other its other components as features of their environment—a search for optimal perspectives and ways to use existing interfaces. At the organism level, an excellent example is the brain, in which an immense number of functions are occurring simultaneously. Interestingly, it has been suggested that the ability to store multiple memories in the same neuronal real-estate is implemented by phase [5].

The results of our probing neural networks for novel functions also suggest that, alongside tools for predicting ways to rewire living systems [240,241,242], we should be developing tools to identify the optimal perspectives with which to view and exploit existing polycomputing capacities.

## 5. Conceptual Transitions

To develop such tools, we will need to overcome human cognitive bias and resist the temptation to cleave the phenomena apart in ways that feel comfortable. One approach is to look for particularly non-intuitive phenomena that defy our attempted categories. Better yet is to seek gradients, along which we can move from the “obvious” approximations of phenomena to increasingly “non-obvious”, but more accurate, reflections of reality.

### 5.1. Directions of Conceptual Travel

One such gradient is the one that leads from serial to parallel to superposed processes. The industrial revolution demonstrated the advantage of performing tasks in parallel rather than serially; the computer age similarly demonstrated the power of parallel over serial computation. One reason for these slow transitions may be cognitive limitations: despite the massive parallelism in the human brain, human thinking seems to proceed mostly, or perhaps completely [243], in a serial fashion. “Traditional” parallelism, as it is usually understood, assumes that multiple processes are coincident in time but not in space. An even more difficult of a concept to grasp is that of superposition: the performance of multiple functions in the same place at the same time.

Another conceptual direction that leads from obvious into non-obvious territories is that which leads from modular processes into non-modular ones. In general, the cardinal rule in engineering, and software engineering in particular, is modular design. However, this is a concession to human cognitive limits, not necessarily “the best way to do things”: many natural phenomena are continua. Taking another step, if we consider biological or technological polycomputing systems, we might ask whether they are modular. However, if a system polycomputes, different observers may see different subsets of functions and some may be more modular than others. In that case, the question of whether a given polycomputing biological system (or bioinspired technology) is more or less modular becomes ill-defined. We argue that, to facilitate future research, these classical distinctions must now be abandoned (at least in their original forms).

### 5.2. Practical Implications for AI/Robotics

Learning how biological systems polycompute, and building that learning into technology, is worth doing for several practical reasons. First, creating more computationally dense AI technologies or robots may enable them to act intelligently and thus, do useful, complex work, using fewer physical materials and thus creating less waste. Second, the technological components that polycompute may be more compatible with naturally polycomputing biological components, facilitating the creation of biohybrids. Third, creating machines that perform multiple computations in the same place at the same time may lead to the creation of machines that perform different functions in different domains—sensing, acting, computing, storing energy, and releasing energy—in the same place at the same time, leading to new kinds of robots. Fourth, polycomputing may provide a new solution to catastrophic interference, a ubiquitous problem in AI and robotics, in which an agent can only learn something new at the cost of forgetting something it has already learned. A polycomputing agent might learn and store a new behavior at an underutilized place on the frequency spectrum of its metamaterial “brain” better than a polycomputing-incapable agent that must learn and incorporate the same behavior into its already-trained neural network controller. Such an ability would be the neural network analogue of cognitive radio technologies, which constantly seek underutilized frequency bands from which to broadcast [244].

## 6. Gradual Computing in Biology: When Does the (Digital) Soul Enter the (Analog) Body?

The importance of continuous models (and the futility of some binary categories) is readily apparent when tracking the slow process of the emergence of specific features that we normally identify in their completed state, and when considering a spectrum of hybrid cases that are readily produced via evolution or bioengineering. Examples include pseudo-problems like “when does a human baby become sentient during embryogenesis”, “when does a cyborg become a machine vs. organism?”, and “when does a machine become a robot?”; all of these questions force arbitrary lines to be chosen that are not backed up by discrete transitions. Developmental biology and evolution both force us to consider gradual, slow changes to be essential to the nature of the important aspects of the structure and function. This biological gradualism has strong parallels in computer science. An unfertilized human oocyte, mostly amenable to the “chemistry and physics” lens, eventually transforms into a complex being for whom behavioral and cognitive (and psychotherapeutic) lenses are required. What does the boot-up of a biologically embodied intelligence consist of? What are the first thoughts of a slowly developing nervous system? One key aspect of this transition process is that it involves polycomputing, as structural and physiological functions become progressively harnessed toward new, additional tasks for navigating behavioral spaces, in addition to their prior roles in metabolic, physiological, and other spaces [22,245,246,247,248]. These ideas also have implication for niche construction and the extended phenotype, in blurring the distinctions between internal and external affordances [249].

Similarly, one can zoom into the boot-up process when a dynamical system consisting of electrical components becomes a computer. During the first few microseconds, when the power is first turned on, the system becomes increasingly more amenable to computational formalisms, in addition to the electrodynamics lens. The maturation of the process consists of a dynamical mode which can profitably be modeled as “following an algorithm” (taking instructions off a stack and executing them). Similarly, one could observe externally supplied vibrations spreading through a metamaterial and consider when it makes sense to interpret the material’s response as a computation or the running of an algorithm. In essence, the transition from an analog device to a computer is really just a shift in the relative payoffs for two different formalisms from the perspective of the observer. These are readily missed, and an observer that failed to catch the ripening of the computational lens during this process would be a poor coder indeed, relegated to interacting with the machine via Maxwell’s laws that guide electron motion and atomic force microscopy, not by exploiting the incredibly rich set of higher-level interfaces that computers afford.

### 6.1. Agency and Persuadability: Implication for Polycomputing

One of the most important next steps, beyond recognizing the degree to which certain dynamical systems or physical materials can be profitably seen as computational systems, is to observe and exploit the right degree of agency. Systems vary widely along a spectrum of persuadability [2], which can be described as the range of techniques that are suitable for interacting with these systems, including physical rewiring, setpoint modification, training, and language-based reasoning. Animals are often good at detecting agency in their environment, and for humans, the theory of mind is an essential aspect of individual behavior and social culture. Consistent with the obvious utility of recognizing the agency in potential interaction partners, evolution has primed our cognitive systems to attribute the intentional stance quite readily [250,251]. Crucially, making mistakes by overestimating this agency (anthropomorphizing) is no worse than underestimating agency—both reduce the effectiveness of the adaptive interactions with the agent’s world.

### 6.2. The Impact of Observer Frames

So far, we have considered a single human observer of a biological or technological system, how much agency they detect in the system from their perspective, and how they use that knowledge to choose how to persuade it to do something. However, a biological system may have many observers (neighboring cells, tissues, conspecifics, and parasites) trying to “persuade” it to do different things, all at the same time (scare quotes here remind us that we must, in turn, decide to adopt the intentional stance for each of the observers). A polycomputing system may be capable of acceding to all of these requests simultaneously. As a simple example, an organism may provide a computational result to one observer while also providing the waste heat produced by that computation to a cold parasite. Traditional computers are not capable of this, or at least are not designed to do so, but future polycomputational machines might be.

### 6.3. Becoming a Computer

For many outside the computational sciences, “computer” denotes the typical physical machines in our everyday lives, such as laptops and smartphones. Turing, however, provided a formal definition for computers that is device-independent: in summary, a system is a computer if it has an internal state, if it can read information from the environment (i.e., its tape) in some way, update its behavior based on what it has read and its current state, and (optionally) write information back out to the tape. This theoretical construct has become known as a Turing machine; any physical system that embodies it, including organisms, is formally referred to as a computer. This broad definition admits a wide range of actors that do not seem like computers, including consortia of crabs [252], slime molds [253], fluids [254], and even algorithms running inside other computers [255]. For all of these unconventional computers, as well as for the novel mechanical computing substrates discussed above, it is difficult to tell at which point they transition from “just physical materials” into computers. With continuous dynamical systems such as these, observers may choose different views from which the system appears to be acting more or less like a physical instantiation of a Turing machine.

Even if an observed system seems to be behaving as if it is a Turing machine, identifying the components of that machine, such as the tape or the read/write head, can be difficult. This is a common reason why it is often claimed that organisms are not machines/computers [19,73,256]. Consider an example from the authors’ own recent work [78]: we found that motile multicellular assemblies can “build” other motile assemblies from loose cells. This looks very much like von Neumann machines: theoretical machines that can construct copies of themselves from the materials in their environment. Von Neumann initially proved the possibility of such machines by mathematically constructing Turing machines that built copies of themselves by referring to and altering an internal tape. However, in the biological Turing machines that we observed, there seems to be no tape. If there is one, it is likely not localized in space and time.

This difficulty in identifying whether something is a computer, or at what point it becomes one, is further frustrated by the fact that biological and non-biological systems change over time: even if one view is held constant, the system, as it changes, may seem to act more or less like a computer. Finally, a polycomputing system, because it can provide different computational results to different observers simultaneously, may at the same time present as different computers—better or worse ones, more general or more specialized ones—to those observers. Such behavior would not only foil the question “Is that a computer?”, but would even foil any attempts to determine the time at which a system becomes a computer, or begins to act more like a computer. Zooming out, it seems that as more advanced technology is created, and as our understanding of biological systems progresses, attempts to attribute any singular cognitive self to a given system will become increasingly untenable. Instead, we will be forced, by our own engineering and science, to admit that many systems of interest house multiple selves, with more or less computational and agential potential, not just at different size scales, but also superimposed upon one another within any localized part of the system.

As Hoel points out about the ad hoc status of claiming one single privileged perspective within a system, according to the integrated information theory (IIT) account of consciousness [257]: “…There are so many viable scales of description, including computations, and all have *some* degree of integrated information. So, the exclusion postulate is necessary to get a definite singular consciousness. This ends up being the most controversial postulate within IIT, however.” [99].

We maintain that, as our understanding of polycomputing biological and technological systems increases, it will eventually exclude the exclusion postulate from any attempt to explain human consciousness as a mental module operating within the brain.

## 7. Conclusions

Prior skeptical debates about whether biological systems are computers reflect both an outdated view of computation and a mistaken belief that there is a single, objective answer. Instead, we suggest a view in which computational interpretations are not simply lenses through which diverse observers can all understand a given system in the same way, but indeed that several diverse interpretations of the information being processed by a set of events can simultaneously be useful to different subsystems (observers) at the same time. It is now seen that there is no one-to-one mapping between biological form and function: the high conservation of the biological form and function across evolutionary instances implements a kind of multiple realizability. At the same time, biological components are massively overloaded with regard to polycomputing. Indeed, their competency, plasticity, and autonomy [2,22,258,259] may enable a kind of second-order polycomputing, where various body components attempt to model each other’s computational behavior (in effect serving as observers) and act based on their expected reward, from their perspective. Thus, modern computer engineering offers metaphors much more suited to understand and predict life than prior (linear and absolute) computational frameworks. Not only are biological systems a kind of computer (an extremely powerful one), but they are amazing *polycomputing* devices, of a depth which has not yet been achieved by technology. In this sense, biological systems are indeed different than today’s computers, although there is no reason why the future efforts to build deep, multi-scale, highly plastic synthetic devices cannot take advantage of the principles of biologic polycomputing. A key implication of our view is that that blanket pronouncements about what living or non-living machines can do are worthless: we are guaranteed to be surprised by outcomes that can only be achieved by formulating and testing hypotheses. It is already clear that synthetic, evolved, and hybrid systems far outstrip our ability to predict the limits of their adaptive behavior; abandoning the absolutist categories and objective views of computation is a first step towards expanding our predictive capabilities.

At stake are numerous practical outcomes, in addition to fundamental questions. For example, to make transformative advances in our ability to improve the health in biomedical settings [260], we must be able to control multiple scales of biological organizations which are heavily polycomputing—from cellular pathways to patient psychological states. It is essential to begin to develop computational frameworks to facilitate that kind of control. The ability to construct and model a kind of computational superposition, in which diverse observers (scientists, users, the agent itself, and its various components) have their own model of the dynamic environment, and optimize their behavior accordingly, will also dovetail with and advance the efforts of synthetic bioengineering, biorobotics, smart materials, and AI.
